# Vaccine Development Against Tuberculosis Over the Last 140 Years: Failure as Part of Success

**DOI:** 10.3389/fmicb.2021.750124

**Published:** 2021-10-06

**Authors:** Stefan H. E. Kaufmann

**Affiliations:** ^1^Max Planck Institute for Infection Biology, Berlin, Germany; ^2^Max Planck Institute for Biophysical Chemistry, Göttingen, Germany; ^3^Hagler Institute for Advanced Study, Texas A&M University, College Station, TX, United States

**Keywords:** BCG, immunity, next-generation vaccine, recombinant, tuberculosis, vaccination, VPM1002

## Abstract

The year 2020 was shaped by the COVID-19 pandemic which killed more people than any other infectious disease in this particular year. At the same time, the development of highly efficacious COVID-19 vaccines within less than a year raises hope that this threat can be tamed in the near future. For the last 200 years, the agent of tuberculosis (TB) has been the worst killer amongst all pathogens. Although a vaccine has been available for 100 years, TB remains a substantial threat. The TB vaccine, Bacille Calmette-Guérin (BCG), has saved tens of millions of lives since its deployment. It was the best and only choice available amongst many attempts to develop efficacious vaccines and all competitors, be they subunit vaccines, viable vaccines or killed whole cell vaccines have failed. Yet, BCG is insufficient. The last decades have witnessed a reawakening of novel vaccine approaches based on deeper insights into immunity underlying TB and BCG immunization. In addition, technical advances in molecular genetics and the design of viral vectors and adjuvants have facilitated TB vaccine development. This treatise discusses firstly early TB vaccine developments leading to BCG as the sole preventive measure which stood the test of time, but failed to significantly contribute to TB control and secondly more recent attempts to develop novel vaccines are described that focus on the genetically modified BCG-based vaccine VPM1002, which has become the frontrunner amongst viable TB vaccine candidates. It is hoped that highly efficacious vaccines against TB will become available even though it remains unclear whether and when this ambition can be accomplished. None the less it is clear that the goal of reducing TB morbidity and mortality by 90% or 95%, respectively, by 2030 as proposed by the World Health Organization depends significantly on better vaccines.

## Introduction

In times of COVID-19 with more than four million deaths caused by SARS-CoV2, the peril of tuberculosis (TB) may be ignored by many. COVID-19 became a global threat within less than 3 months after the first outbreak in Wuhan in December 2019. It spread over the globe so rapidly that it was announced as “pandemic” by the World Health Organization (WHO) on March 12, 2020 (Ghebreyesus, 2020). This immediately led to the development and deployment of novel vaccines with the notable example of m-RNA vaccines and adenovirus-vectored vaccines (Subbarao, 2021). With more than 90% protection offered by m-RNA vaccines and more than 80% protection by adenovirus-vectored vaccines the efficacy of these vaccines is excellent. In contrast, the threat of TB has rampaged over the globe for more than a millennium and was the major killer in the capitals of the industrialized world in the nineteenth century (Pai et al., 2016; WHO Global Tuberculosis Report 2020, 2020). A single TB vaccine has been available since 1921, termed Bacille-Calmette-Guérin (BCG) which is, however, far less efficacious than available COVID-19 vaccines (Zwerling et al., 2011). Moreover, the degree of protection afforded by BCG varies in different areas of the world (No Author List, 1979; Colditz et al., 1994, 1995; Zwerling et al., 2011; Mangtani et al., 2014; Roy et al., 2014; Brazier and McShane, 2020). Notably, the majority of regions suffering from high TB prevalence are located around the equator, where BCG seems to be least effective (Wilson et al., 1995). Aggravating the situation, TB prevalence overlaps with HIV infection rates and HIV-infected individuals have a 10-fold higher risk of TB disease rendering it the number one killer of HIV-infected individuals (WHO Global Tuberculosis Report 2020, 2020). In contrast to COVID-19, TB can be cured efficaciously by drug treatment. However, increasing incidences of drug resistance render this option less satisfactory (WHO Global Tuberculosis Report 2020, 2020). Hence, TB will only be brought under control by a combination of better drugs, diagnostics and vaccines. In this treatise I will describe TB vaccine development over the last 140 years. I take the 100th anniversary of the introduction of BCG as reason for devoting much space to the early stages of TB vaccine research and development (R&D) and will then focus on the pipeline of current vaccine candidates in clinical trials emphasizing the genetically upgraded next-generation BCG vaccine, VPM1002.

## Specific Features of Tuberculosis as They Relate to Vaccination Strategies

*Mycobacterium tuberculosis* causes a chronic infection which can, but need not, result in a chronic disease with high lethality if untreated (Gengenbacher and Kaufmann, 2012; Pai et al., 2016). *M. tuberculosis* is transmitted in aerosols and the lung is the major port of entry, the prime site of disease symptoms and the main source of transmission. Infection induces an acquired immune response composed of antibodies and T cells (Ottenhoff and Kaufmann, 2012). General assumption considers T lymphocytes as major mediators of protective immunity. Soon after infection, phagocytes, notably mononuclear phagocytes and neutrophils, enter the site of bacterial growth, where they engulf the mycobacteria. Some microorganisms are killed, whereas others survive persisting inside mononuclear phagocytes. Granulomas are formed which in their well-structured solid stage contain *M. tuberculosis* under the direction of T lymphocytes (Ulrichs and Kaufmann, 2006). CD4^+^ helper-1 T cells (TH1 cells) are considered critical for protective immunity, notably after maturation into memory T cells (Ottenhoff and Kaufmann, 2012). Increasing evidence suggests a role for TH17 cells and for CD8^+^ cytolytic T lymphocytes (CTL) (Ulrichs and Kaufmann, 2006; Ottenhoff and Kaufmann, 2012; Coulter et al., 2017). Also, a role of B lymphocytes and antibodies in TB control, long considered of low to no relevance, has been reinvigorated (Rijnink et al., 2021).

Infection can lead to primary TB disease with insignificant symptoms that are often overlooked before they recede. However, *M. tuberculosis* may persist in the host, causing latent TB infection (LTBI) in about 1.7 billion individuals (Houben and Dodd, 2016). About 90% of these individuals are thought to survive lifelong with LTBI; the remaining ca. 10% will develop active TB disease. Although most cases progress to clinical TB within the first 2 years after infection, reactivation can be delayed over longer periods of time. During LTBI, *M. tuberculosis* is contained in granulomas in a dormant stage in which the bacillus has changed its genetic program allowing for survival in hypoxic surroundings (Gengenbacher and Kaufmann, 2012). These dormant bacteria are difficult to assail by drugs and immune defense. As a consequence of exhausted or otherwise disabled immunity, the granuloma loses its protective functions and becomes increasingly necrotic and later caseous (Ulrichs and Kaufmann, 2006). Whilst this is detrimental to the host, it is beneficial for *M. tuberculosis* which takes the opportunity to “wake up” and grow virtually unrestrictedly (Gengenbacher and Kaufmann, 2012). The bacilli not only replicate in the caseous granuloma, but also spread to other organs and into the environment by coughing and expectoration (Pai et al., 2016). Thus, patients suffering from active TB disease are often contagious. The high prevalence of LTBI, currently estimated to exist in one fourth of the global population, the chronicity of disease as well as the critical role of complex T cell-mediated immunity in control of *M. tuberculosis* furnish several obstacles for development of a vaccine with high protective efficacy.

Next generation vaccines must be better than BCG and need to achieve one or more of the following criteria (Brazier and McShane, 2020; Kaufmann, 2021):

• Prevention of infection (PoI): Such a vaccine does not allow *M. tuberculosis* to stably establish itself in the host, but most likely permits short-term infection which is rapidly terminated.
• Prevention of disease (PoD): Such a vaccine allows stable infection of *M. tuberculosis*, but contains the pathogen thereby preventing TB reactivation. Preferably, but not necessarily, PoD would be accompanied by long-term sterile eradication of *M. tuberculosis*.
• Prevention of recurrence or relapse (PoR): Such a vaccine targets individuals who had been cured from TB disease by drug treatment. In some individuals, *M. tuberculosis* hides in secluded niches thereby evading elimination by drugs. This so-called relapse, therefore, poses a risk for active TB disease. Alternatively, drug treatment is successful in eliminating *M. tuberculosis*, but cured individuals remain vulnerable to reinfection. PoR due to either relapse or reinfection represents a target for next-generation vaccines.

Another benchmark is the time of vaccine administration relative to infection (Kaufmann, 2021). BCG is approved as a pre-exposure vaccine given to neonates as soon as possible after birth. Hence, novel vaccines to replace BCG are pre-exposure vaccines administered before infection with *M. tuberculosis*. Aside from neonates, this group also includes uninfected adolescents and adults. In the latter case, vaccination can build on previous BCG immunization in a prime/boost vaccination schedule. In sum, these vaccines induce PoI and/or PoD. The large number of individuals with LTBI necessitates the development of next-generation vaccines which are given post-exposure with *M. tuberculosis*. Obviously, these vaccines cannot induce PoI and have to attain PoD, either accompanied by sterile elimination or long-term *M. tuberculosis* containment. Strictly speaking, vaccines for PoR are post-exposure vaccines. Given that they can prevent relapse, they have to either contain or eliminate *M. tuberculosis*. Alternatively, they have to induce an immune response better than the one which failed to contain primary infection in order to prevent reinfection.

## Discovery of *M. Tuberculosis*

**FIGURE 1:**
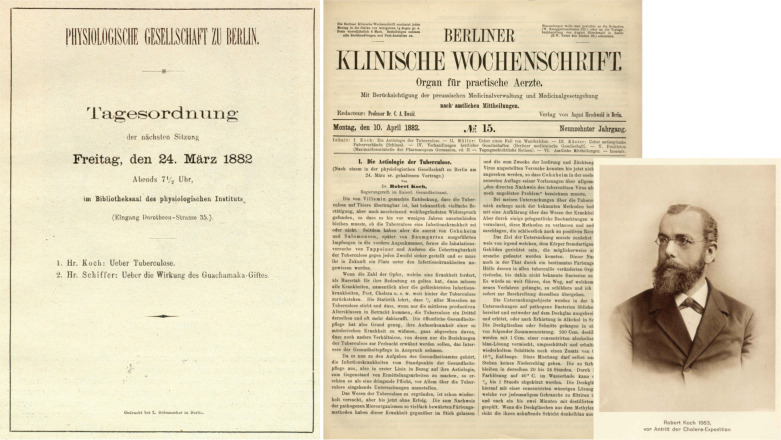
Description of the etiology of TB by Robert Koch. Agenda of the meeting of the Physiological Society in Berlin, where Robert Koch for the first time presented his data on March 24, 1882 and first page of the publication on this topic in the Berlin Clinical Weekly, April 10, 1882 (Koch, 1882). Figure also includes photo of Robert Koch from 1883 (Kaufmann and Winau, 2005).

**FIGURE 2:**
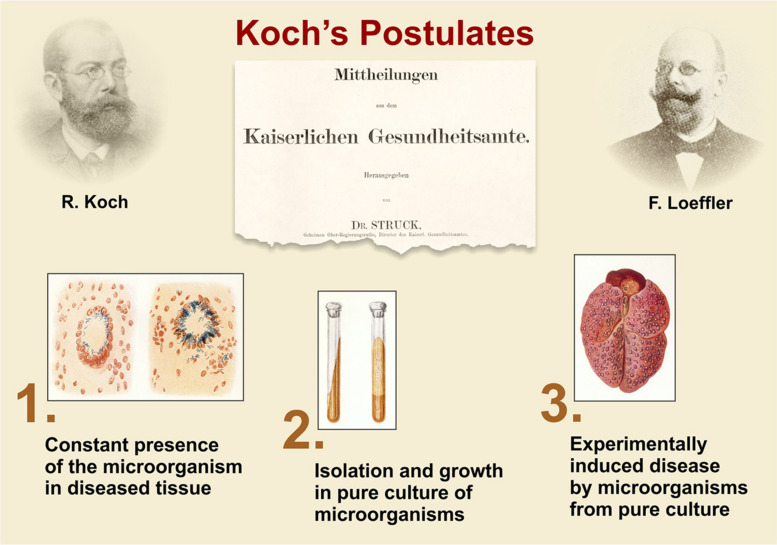
Koch’s postulates, based on Robert Koch’s lecture on the etiology of TB and phrased in general terms by F. Loeffler (Loeffler, 1884; Kaufmann and Winau, 2005).

The discovery of the etiologic agent of TB by Robert Koch (1843–1910) formed the basis for rational understanding of the disease and served as a platform for design of novel intervention measures including diagnostics, vaccines and therapeutics. In his groundbreaking description of the etiology of TB, first orally on March 24, 1882, at a meeting of the Physiological Society in Berlin and less than 3 weeks thereafter in written form in the Berlin Clinical Weekly published April 10, Koch carefully described his work (Figure 1; Koch, 1882; Kaufmann and Winau, 2005). His strategy later led to the formulation of the so-called Koch’s postulates by Friedrich Loeffler which comprise (Figure 2; Loeffler, 1884; Kaufmann and Winau, 2005):

• Constant presence of the microbe in question in diseased tissue;
• Isolation and growth on solid culture of the responsible microbe;
• Experimental induction of a similar disease by the isolated microbe from pure culture.

In fulfillment of these postulates, a unique type of pathogenic microorganism specifically causes a unique disease with characteristic symptoms. This ultimately allowed dismissal of other theories of infectious disease etiology (Kaufmann and Winau, 2005). First, it clarified unequivocally that certain diseases are caused by exogenous invaders, not by malfunctioning host cells as proposed by the pathologist Rudolf Virchow. Second, it dismissed the belief that transmission of such diseases was caused by a miasm, a belief dating back to Hippocrates and strongly favored by Max von Pettenkofer, a contemporary of Koch. Third, it dismissed the notion that a single disease can be caused by various types of microbes and vice versa that a single microbe can cause different types of disease through transformation into new variants as e.g., proposed by Carl Wilhelm von Naegeli.

In his groundbreaking studies, Koch meticulously analyzed human granulomas as well as lesions from different animal species including cattle, pigs, chicken, monkeys, guinea pigs and rabbits. In all cases the characteristic bacilli were identified by appropriate staining methods. Lesions were mostly present in the lung, but Koch also isolated bacteria from lesions in brain, intestine, lymph nodes from swollen nodes in necks (as characteristic signs of scrofulosis) and joints (known to cause arthritis). Proof that disease could be replicated in experimental animals by a pure culture of bacilli was based on infection studies with guinea pigs, rabbits and cats. As extraordinary as this paper was, it contained one major error which Koch would not accept for almost 20 years. Since he had identified the TB bacilli in diseased cattle, he insisted that bovine TB (perlsucht) and human TB (phthisis or scrofulosis) were caused by the same pathogen. It took him until 1901 to accept that human and bovine disease were caused by different agents, the former one being *M. tuberculosis*, the latter *Mycobacterium bovis* (Koch, 1912a). He based his conclusion on his own studies although in other countries, notably the United States, United Kingdom and France, it was already generally accepted that distinct types of mycobacteria were responsible for human and bovine disease. Most credit goes to Theobald Smith for providing compelling evidence that the two diseases were caused by distinct mycobacteria (Smith, 1898).

Even after his lecture in 1901, Koch dismissed *M. bovis* as frequent cause of TB in humans (Koch, 1912b). Yet, numerous researchers already considered *M. bovis* a major cause of infant TB. They reasoned that intestinal TB was primarily due to ingestion of *M. bovis* contaminated milk and therefore diagnosis of intestinal TB was often considered equivalent to *M. bovis* infection. Koch’s former disciple, Emil von Behring, went one step further and claimed that a high proportion of adult TB was due to reactivation of childhood infection with *M. bovis* following gut infection (Behring, 1915).

## Koch’s Attempt to Immunize Against Human Tuberculosis

Koch was also the first to claim that he had developed a vaccine against TB. In 1890 at the 10th International Medical Congress in Berlin, in his introductory speech he implied that he had discovered a remedy to both prevent and cure TB (Koch, 1890a).

His claims were rather vague and regarding preventive vaccination he claimed that:

“… guinea pigs which are extremely susceptible to tuberculosis do not respond to challenge with the tubercle bacillus when pretreated with this remedy …” and further referring to efficacious treatment in response to his remedy that “ … in guinea pigs which already suffer from severe systemic tuberculosis, the disease process can be arrested by this remedy without major side effects …”.

**FIGURE 3:**
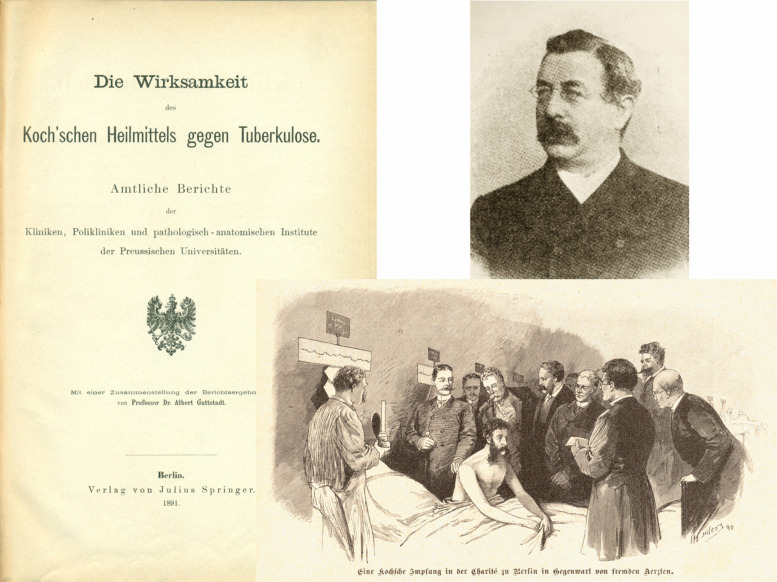
Report describing the clinical trials with Koch’s remedy against TB. The figure shows title page of report and photo of the principle investigator (Guttstadt, 1891; Kaufmann and Winau, 2005). Also shown is a picture from a magazine depicting vaccination of a TB patient with tuberculin in front of medical doctors.

Koch became a hero of medicine and his scientific talk was soon widely publicized in newspapers and magazines (Kaufmann and Winau, 2005). Consequently, the remedy was soon widely harnessed for treatment of TB patients coming to Berlin from all over the world in hope of a cure of this scourge (Figure 3). Whilst this all happened within weeks, the composition of the remedy remained secret until a first description appeared later that year (Koch, 1890b). Yet, even then, the exact composition remained vaguely defined and only the second publication in 1891 described it as a glycerin extract from pure cultures of tubercle bacilli (Koch, 1891a). Finally, in his third publication on this topic, information on production and quality testing was revealed. In these studies, the remedy was termed “tuberculin” (Koch, 1891b).

We now know that tuberculin is primarily composed of proteinaceous antigens and various glycolipids. In more modern terms, it could be described as a subunit vaccine containing proteins as antigens for antibodies and conventional T cells and glycolipids as antigens for unconventional T cells and antibodies, but also acting as adjuvant. In 1890, a clinical study to test tuberculin as a therapeutic vaccine was initiated. This was a multicenter study of efficacy and safety under the leadership of Albert Guttstadt (Guttstadt, 1891). The report appeared in 1891 summarizing results from more than 1750 patients (Figure 3). A total of 1,061 patients suffered from TB of internal organs, notably the lung, whereas 708 patients had TB of external tissues including bones and joints. The results were extremely disappointing. Amongst patients suffering from internal TB, only 1.2% were considered cured and amongst patients with external TB 2.1%. Even though a relatively high proportion, namely 34% of patients with internal TB and 54% of patients with external TB apparently showed signs of improvement, this data was confounded by high subjectivity. Although control groups were missing, experience had shown that these effects were to be expected in patients receiving canonical clinical care. In sum, the trial did not provide evidence for therapeutic effects of tuberculin against TB. Because of these disappointing findings, tuberculin was never tested as a preventive vaccine. Tuberculin, however, was soon found to be a valuable diagnostic tool because when given subcutaneously to TB-infected individuals, it caused a characteristic skin reaction within 2–3 days, now called delayed-type hypersensitivity reaction (Duthie and Reed, 2021). Yet, the tuberculin skin test (TST) does not distinguish between healthy individuals with LTBI and patients with active TB disease. A more refined version of tuberculin—purified protein derivative (PPD)—is used until today for diagnosis of *M. tuberculosis* infection. In fact, the TST [or its more modern versions, the Interferon-γ release assays (IGRA)] form the basis for the assumption that one third to one fourth of the world population is infected with *M. tuberculosis* (Houben and Dodd, 2016).

In conclusion, the first attempt to develop a TB vaccine was a complete disaster, but paved the way for subsequent endeavors to develop a vaccine against this threat.

## Different Approaches Toward Development of a Tuberculosis Vaccine

The 30 years between 1890, when Koch had first announced his vaccine against TB, and 1921, when Albert Calmette (1863–1933) and Camille Guérin (1872–1961) administered BCG to a human neonate for the first time, were characterized by the development and clinical testing of different vaccine types including subunit vaccines, inactivated vaccines and live whole-cell vaccines with only one final success: BCG (Calmette et al., 1927). This vaccine was created following the principles of Pasteur to attenuate the causative agent of disease by culture conditions which caused loss of virulence factors. This was not the only approach based on live vaccines. Other attempts employed minute doses of *M. bovis* or *M. tuberculosis* themselves to immunize cattle or humans, respectively (Moeller, 1903, 1904; Koch et al., 1905; Smith, 1911; M’Fadyean et al., 1913; Rabinowitsch, 1913; Behring, 1915). They followed the concept of variolation widely used on the Asian continent and propagated by Lady Montagu in Western Europe prior to the introduction of Jenner’s cowpox (Montagu, 1790; Jenner, 1798).

A widely tested approach was based on the assumption that *M. tuberculosis* was relatively harmless when given to cattle. Hence, this approach was considered for prevention of bovine disease in the veterinary field by Behring and Koch in Germany, by Smith in the US, and by M’Fadyean in UK amongst others (Koch et al., 1905; Smith, 1911; M’Fadyean et al., 1913; Behring, 1915). Behring’s vaccine, the Bovovaccine, showed evidence for some short-term protection but ultimately failed (Behring, 1915). Koch’s Tauruman using *M. tuberculosis*, cultured in such a way that it partially lost virulence, was disappointing, and many calves died directly after immunization (Koch et al., 1905). In sum, all these approaches were unsuccessful.

Reciprocally, different mycobacteria were considered as vaccines against human TB including *M. bovis* and mycobacteria from birds or from cold-blooded animals (Friedmann, 1903, 1904; Moeller, 1903, 1904; Wells, 1937; Wells and Brooke, 1940). Immunization against human TB using *M. tuberculosis*, passaged through cold-blooded animals, was initially propagated by Friedmann and Moeller, but soon found to be totally useless. Moeller and Friedmann, however, did not give up and harnessed mycobacteria directly isolated from cold-blooded animals. Moeller had derived his mycobacteria from blindworms and Friedmann from turtles (Friedmann, 1903, 1904; Moeller, 1903, 1904; Calmette et al., 1927). Intriguingly, these mycobacteria could be cultured at 25°C, but did not replicate at 37°C. Both vaccines were commercialized, but it was soon established that experimental animals immunized with these live vaccines did not survive challenge with *M. tuberculosis* (Uhlenhuth and Lange, 1920). These approaches followed Jenner’s strategy, who in 1796 had published his successful vaccination against smallpox using cowpox (Jenner, 1798).

Vaccinations with heat-killed M. tuberculosis were tested both for cattle and human TB by numerous scientists including Calmette and Guérin in France (Calmette et al., 1927), Smith in the US and Loeffler in Germany (reviewed in Calmette et al., 1927). Because these simple attempts all failed, further refinements were introduced by different chemical treatments to not only kill, but also split the bacilli. Vaccines based on components of *M. tuberculosis* included enzymatic digestion of *M. tuberculosis* by proteases and lipases (Levy et al., 1908; Loeffler, 1913). Deycke and Much used neurine and choline and later bovolecithin and in another preparation weak acids, such as lactic acid for inactivation. They hoped these treatments would lead to the enrichment of a selected group of antigens—which they termed “partigens”—that would induce protection (Deycke and Much, 1909, 1913). Others attempted to attenuate the bacilli by treatment with chemical compounds rich in chlorine, iodine and fluorine (Calmette et al., 1927). All without success. Calmette and Guérin tried boiling with acetone, ether, carbon tetrachloride methyl, alcohol or petroleum ether to extract lipid-rich components from *M. tuberculosis* (Calmette et al., 1927). Indeed, in some cases this led to preparations of reduced virulence which still induced antibody responses; yet protection was not achieved.

Following their unsuccessful attempts to develop a protective vaccine by chemical treatment and encouraged by a certain degree of protection (although with severe adverse events) by immunization with minute doses of live *M. tuberculosis* in cattle, Calmette and Guérin decided to develop a live vaccine against human TB by attenuating *M. bovis* through culture under virulence-reducing conditions (Calmette, 1922, 1931; Calmette et al., 1927). They had already observed that treatment of *M. bovis* with ox bile reduced virulence. The risk of reversion to virulence in the host however remained after short-term culture and, hence, long-term serial passage was required. Moreover, their experience taught them that oral administration was probably the most appropriate route of vaccination.

## The Bacille Calmette-Guérin Vaccine

**FIGURE 4:**
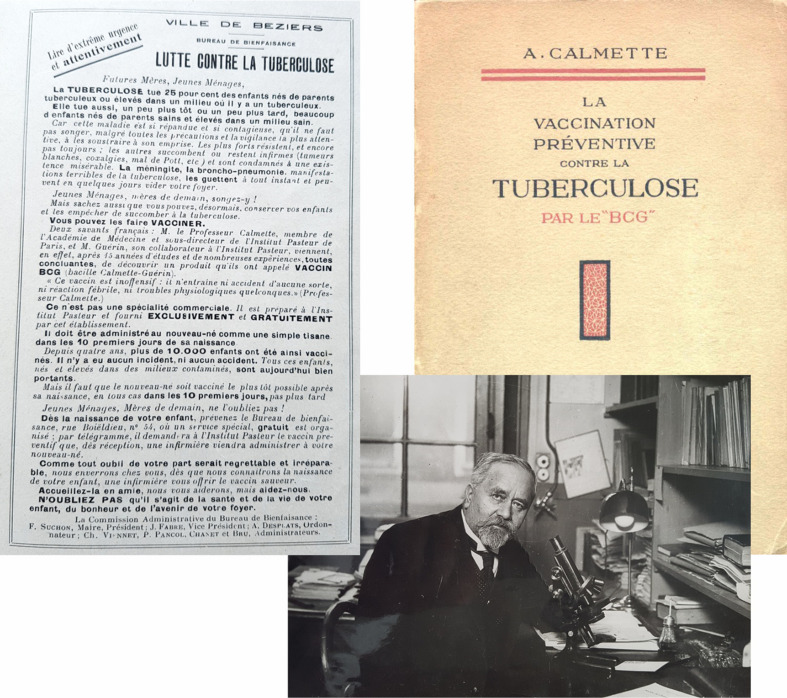
Description of development and clinical testing of BCG. Figure shows title page of book, photo of the author, Albert Calmette, and leaflet advocating BCG vaccination of neonates from the book (Calmette et al., 1927).

Calmette and Guérin reached the conclusion that subunit and killed whole cell vaccines would not induce sufficient protection and that harmful components removed from viable vaccines would be rapidly reestablished in the host because the attenuation was instable. They decided that a stable, attenuated live vaccine would be the only way forward. This led to the development of Bacille Bilie Calmette-Guérin, later termed Bacille-Calmette-Guérin (in short BCG) between 1906 and 1921 (Figure 4; Calmette, 1922, 1931; Calmette et al., 1927). This vaccine was targeted primarily at newborns to reduce childhood mortality due to TB. Hence, the approach of Calmette and Guérin is a combination of strategies fostered by Jenner and Pasteur (Jenner, 1798; Pasteur, 1881). Following Jenner’s strategy, they used the causative agent of cattle TB, *M. bovis*, against human TB and following Pasteur’s strategy they cultured the bacteria under attenuating conditions. Notably, they introduced long-term serial passage to achieve stable attenuation essential for a TB vaccine. Treatment of *M. bovis* on potato slices soaked with ox bile and glycerin and dipped into culture broth led to a fine dispersion of microbes virtually devoid of clumps. Under these conditions, the bacteria grew over 20–25 days and then had to be transferred to the next culture. This was continued between 1908 and 1920 until 230 passages had been completed. The resulting strain was found innocuous for a variety of animal species naturally susceptible to *M. bovis*. Moreover, the strain induced protection in cattle against challenge with wild-type *M. bovis* infection lasting for longer than a year. These experimental animal studies were then enlarged to field experiments which showed that the vaccine given to newly born calves could reduce bovine TB even at farms where the disease was prevalent (Calmette et al., 1927). It also appeared that multifold oral administration of the vaccine was most successful.

Parallel studies revealed that high doses of more than 10^7^ bacilli were safe in guinea pigs, rabbits and various non-human primates even after intravenous application (Calmette et al., 1927). In July 1921, Calmette and Guérin together with B. Weill-Hallé performed the first vaccination of a newborn in a household, where the mother had died and the grandmother, who raised the infant, suffered from active TB (Calmette et al., 1927). The neonate received three oral doses of 2 mg of BCG each amounting to a total of approximately 2.4 × 10^9^ bacilli suspended in milk. (As a reminder: the current BCG vaccine contains 10^6^ bacilli given as a single intradermal dose.) Over the whole observation period of 5.5 years, this infant never developed TB disease. Following immunization of 120 infants over the next year, the vaccination protocol was slightly changed to three oral applications every second day over the first 10 days after birth with 1 mg each amounting to a total of 1.2 × 10^9^ bacilli. By early 1927, a total of 21,200 infants had been vaccinated with BCG. Of the 120 neonates vaccinated in the first year, 80 babies were followed up for 4 years and found to be healthy. Twenty-four of them had lived in a household with at least one TB patient. Of the 21,200 neonates vaccinated till February 1st, 1927, 969 children were followed-up because they were living for at least 1 year in a TB household after vaccination. A controlled study was not performed and therefore Calmette and Guérin based their efficacy calculations on the assumed death rate of infants born in a household with active TB in different areas of France. Considering an average of 25% death rate of such babies, they came to the following conclusion: TB mortality of vaccinated infants was ca. 1% and general mortality was 8.7%.

Notably the impact on general mortality was unexpected and led Calmette to ask the question (Calmette, 1931): “How can this difference between general mortality in vaccinated and in non-vaccinated groups be explained?”

He offered two alternative explanations which he phrased as questions:

First: “… can it be that tuberculosis infection plays a more important part in infant mortality than we have supposed?”

Second: “…does the harboring of BCG followed by its digestion and elimination confer on the organism a special aptitude to resist those other infections which are so frequent in young children?”

Although these were impressive data, the high proportion (25%) of TB death amongst those chosen as unimmunized controls was criticized by others. They interpreted a marked protection against general mortality as evidence that BCG was mostly used in selected populations where general mortality of neonates was lower due to better living conditions as compared to the total population (Greenwood, 1928; Rosenfeld, 1928; Freudenberg, 1930; Wolff, 1930). Today we know that BCG induces heterologous protection against other infections in low income countries likely due to training of innate immunity afforded by macrophages (Aaby and Benn, 2019; Netea et al., 2019). Nevertheless, the arguments of statisticians against comparison of two largely unrelated groups in an uncontrolled way made a valid point, notably because the assumption of 25% deaths in untreated babies was derived from earlier epidemiologic studies and the percentage had probably declined in the years between 1921 and 1927 (Greenwood, 1928; Rosenfeld, 1928; Freudenberg, 1930; Wolff, 1930). Another point of critique was the finding that intravenous inoculation of very high doses into experimental animals caused the formation of lesions which was taken as evidence for residual virulence (Malkani, 1930). Moreover, a major point of critique focused on the risk of reversion to virulence and indeed several publications stated that they had observed reversion to virulence of BCG in experimental animal studies (Dreyer and Vollum, 1931; Petroff, 1931). We now know that during attenuation, BCG lost a gene cluster present in *M. bovis* and *M. tuberculosis* known as. region of difference 1 (RD-1) that encodes major virulence factors including ESAT-6 and CFP-10 (Mahairas et al., 1996; Behr and Small, 1999; Behr et al., 1999; Pym et al., 2002). It is extremely unlikely that the whole gene cluster encoding nine open reading frames could be reintegrated into the genome of BCG by a “gain of function experiment of nature.” Finally, prevention of death was considered an incomplete clinical endpoint and inclusion of prevention of disease was considered essential.

Today BCG is the most widely applied vaccine globally with > 4 billion doses administered in total with ca. 120 million vaccinations per year through the Expanded Program of Immunization (WHO Global Tuberculosis Report 2020, 2020). Although Calmette and Guérin had propagated oral vaccination of neonates, they also tested subcutaneous vaccination which they found less appropriate (Calmette et al., 1927; Albrecht and Paul, 1950). Intradermal vaccination as is mostly done nowadays was introduced by Wallgren in 1928, who had observed 100% TST conversion after intradermal versus 40–85% TST conversion after oral administration (Wallgren, 1928; Albrecht and Paul, 1950; Sjögren, 1982). Also, longevity of TST conversion was maintained longest after intradermal application with more than 80% positivity after 5 years in contrast to 50% loss of TST conversion within a year after oral application (Wallgren, 1928; Albrecht and Paul, 1950; Sjögren, 1982).

## The Lubeck Disaster

By 1927, BCG had already been distributed to 28 countries in Europe, the Americas and Middle East. The vaccine was produced at the Pasteur Institute and was handed out freely under strict regulations (Figure 4; Calmette et al., 1927). Whilst BCG vaccination was increasingly performed in numerous countries outside France, including Belgium, Netherlands, Rumania, and Poland, it was not recommended in Germany. Although a group of experts at the League of Nations (the predecessor of the United Nations) categorized BCG vaccination as harmless, the German Health Office recommended to not test BCG in humans and to await additional experimental investigations. Yet, a few practitioners in Germany had already started BCG vaccination. And in 1929, the director of the local Health Office in Lűbeck in northern Germany, Ernst Altstaedt, and the director of the local General Hospital, Georg Deycke, decided to organize a large BCG vaccine campaign (Science Reports, 1930; American Journal of Public Health and the Nation’s Health, 1931; Moegling, 1935). Deycke together with Much had already attempted to develop a subunit vaccine against TB based on “partigens,” but in vain (see above) (Deycke and Much, 1909; Deycke and Much, 1913). In July 1929, a BCG culture was requested from the Pasteur Institute in Paris and received a few weeks later. The relevant decision makers approved the vaccination campaign which started late February, 1930. Based on a French BCG leaflet (Figure 4), a call was published in local newspapers encouraging parents to have their newborn immunized with BCG. In striking contrast to the French version, neither the call nor the handouts given to parents in Lűbeck mentioned the term “immunization with a live vaccine.” Rather it described the process as “feeding of Calmette’s remedy.”

**FIGURE 5:**
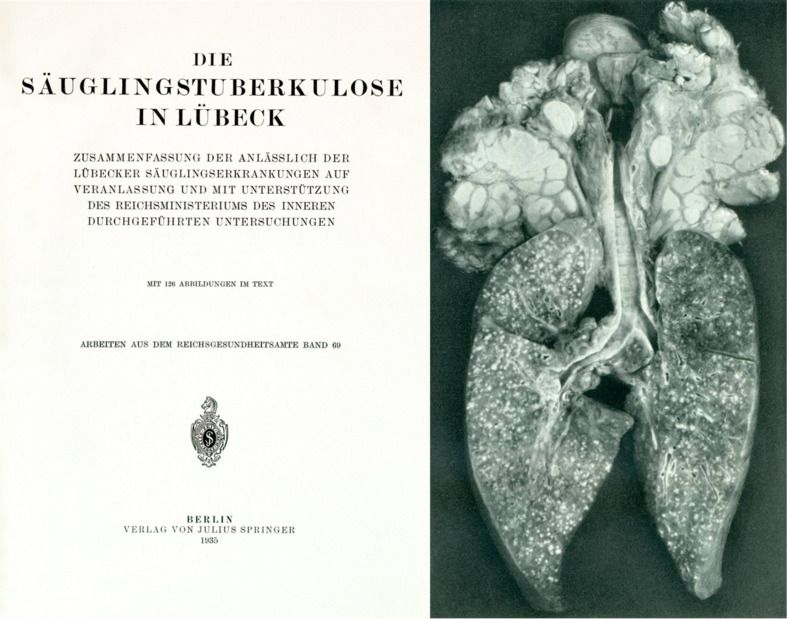
Title page of the report on investigations into the so-called Lubeck disaster and photo from this report depicting lung and lymph nodes with numerous lesions caused by *M. tuberculosis* contamination of BCG (Moegling, 1935).

More seriously, the precautions requested by Calmette and Guérin were not followed: firstly appropriate animal experiments to validate the lack of virulence of the vaccine were not undertaken and secondly the laboratory where the vaccine was prepared was also used for culture of recently received virulent *M tuberculosis* bacilli (Calmette et al., 1927). This strain produced unusual green pigments which later helped to identify contamination of the BCG culture with the tubercle bacillus (Moegling, 1935). Between February and May, 1930, 251 of 412 babies born during this period received the contaminated vaccine, but were not routinely followed-up. After the first infants had died in April, Deycke stopped the vaccination campaign and destroyed most of the vaccine phials. Yet, some vaccinations were still performed because the medical staff was insufficiently informed. Finally, on May 6th the Lubeck Health Office informed the hospitals and the public as well as the National Health Office regarding the disaster, asking for an official investigation by an expert committee (Moegling, 1935). Their conclusion unambiguously stated (Figure 5):

• Cases of disease and death were not due to BCG.
• The cause of the disaster was most likely contamination of BCG with *M. tuberculosis*.

Of the 251 vaccinated babies 41 did not develop any symptoms of TB. Of the 210 babies developing TB, 75 died of or with TB (Moegling, 1935). Deycke and Altstaedt were convicted of negligently causing the neonates’ bodily harm and death. Even though it was clear that the catastrophe was due to a contamination of the vaccine, the consequences for BCG acceptance were serious. It took until 1947 before BCG vaccination was widely offered in Germany on a voluntary basis (Albrecht and Paul, 1950).

## From Mycobacteria Contaminated Butter to Complete Freund’s Adjuvant

One of the most potent experimental adjuvants is complete Freund’s adjuvant composed of a mineral oil and killed mycobacteria and applied as water-in-oil emulsion (Opie and Freund, 1937; Freund, 1947, 1951; Finger, 1964). Although it is rarely used nowadays due to severe adverse events, it has helped numerous immunologists perform their experiments by inducing strong cell-mediated immune responses. As the name indicates, the development of this adjuvant is generally credited to Jules Freund, but first evidence stems from the work of Lydia Rabinowitsch (1871–1935) who had studied mycobacterial contaminations in milk products, notably butter (Rabinowitsch, 1897). Between 1894 and 1898, she worked both in Germany at the Institute of Robert Koch in Berlin and in the US at Women’s Medical College in Philadelphia. She mostly focused on mycobacterial contamination of milk and milk products. In her earlier studies, she had identified an atypical mycobacterium in butter which could be definitely distinguished from *M. bovis* and *M. tuberculosis* and was later called *Mycobacterium butyricum*. In an effort to determine whether this mycobacterium would cause disease, she applied it to a variety of experimental animals and found it rather harmless. Intriguingly, however, she observed that application of mycobacteria in butter induced strong local reactions which resembled lesions caused by *M. tuberculosis* (Rabinowitsch, 1897). Later it was shown by Grassberger that this effect was even stronger when other oily substances including paraffin oil were used instead of butter (Freund, 1951; Grassberger, 1899). Between 1898 and 1903, Rabinowitsch held an independent research position at Koch’s Institute and identified *M. bovis* in some batches of butter from the largest dairy in Berlin. She could trace this back to dairy cows with perlsucht (Rabinowitsch, 1899). Building on studies of Ramon, Glenny and others that certain compounds were able to induce stronger immune responses against antigens, Freund and colleagues developed the Freund’s adjuvant, first using killed *M. tuberculosis* and later atypical mycobacteria such as *M. butyricum* together with mineral oil (Ramon, 1923; Glenny et al., 1926; Opie and Freund, 1937; Freund, 1947, 1951; Finger, 1964; Kaufmann, 2019b).

## From Bacille Calmette-Guérin to Novel Vaccine Candidates

With increasing deployment of BCG it soon proved its value for neonatal vaccination; yet, later its drawbacks became increasingly clear (Albrecht and Paul, 1950; Lange et al., 2021). Today it is fair to state that BCG protects against extrapulmonary TB as it frequently occurs in infants, but is insufficient against pulmonary disease in all age groups, notably adolescents and adults (Colditz et al., 1994, 1995; Kaufmann, 2021; Mangtani et al., 2014; Roy et al., 2014). Hence, BCG vaccination has little impact on incidences of TB morbidity and mortality because *M. tuberculosis* infection is primarily spread by aerosols expelled by adult patients with pulmonary TB. With the advent of effective TB drug treatment, interest in developing better TB vaccines ceased, not the least because of negligible funding (Kaufmann and Parida, 2007). Increasing incidences of resistance ranging from single via multi to extreme drug resistance emphasized the urgent need for better prevention measures (WHO Global Tuberculosis Report 2020, 2020). Today it is clear that TB can only be conquered if we have better vaccines, drugs and diagnostics at hand. This was clearly emphasized at the high-level meeting on TB convened by the United Nations on September 26th, 2018, where it was stated: “…. [we] reaffirm our commitment to ending the TB epidemic globally by 2030 …” and “… that TB including its drug resistant forms is a critical challenge and the leading cause of death from infectious diseases, the most common form of antimicrobial resistance globally and the leading cause of death of people living with HIV …” (United Nations [UN], 2018). Finally, the urgent need for new interventions was voiced as a commitment: “… to create an environment conducive to research and development of new tools for tuberculosis, and to enable timely and effective innovation and affordable and available access to existing and new tools and delivery strategies …” (United Nations [UN], 2018).

Even though 2020 witnessed the dramatic health threat by COVID-19 leading to morbidity and mortality rates that exceeded those of TB, it is likely that in the near future TB will regain its inglorious lead position of the major killer amongst all infectious agents (Stop Tb Partnership Civil society-led Tb/Covid-19 Working Group, 2020; McQuaid et al., 2021; World Health Organization [WHO], 2021). COVID-19 has dramatically impacted on TB notification rates with serious consequences for TB control. According to one estimate, COVID-19 will cause an excess of 1.4 million TB deaths and 6.3 million cases of active TB by 2025 (Stop Tb Partnership Civil society-led Tb/Covid-19 Working Group, 2020). Hence, better TB interventions are needed more urgently than ever before, if we want to reach the goal of controlling TB by 2030. It is hoped that the successful R&D and the virtually unlimited funding, for COVID-19 vaccines will provide helpful guidance for improved TB R&D from preclinical to clinical studies followed by accelerated deployment.

Toward the end of the twentieth century, initiatives to develop vaccines that can replace, or improve on, BCG increased (Kaufmann et al., 2017; Andersen and Scriba, 2019; Ginsberg, 2019; Brazier and McShane, 2020; TuBerculosis Vaccine Initiative [TBVI], 2020; Kaufmann, 2021). As in the thirty years prior to the introduction of BCG, these include subunit vaccines, as well as inactivated and viable whole cell vaccines. A new addition are viral vectored vaccines expressing defined Mtb antigens (Smaill et al., 2013; Tameris et al., 2013; Stylianou et al., 2015). Indeed, the first vaccine candidate tested for protective efficacy against TB in a clinical phase IIb trial was an MVA vector vaccine expressing an *M. tuberculosis* antigen (MVA = Modified Vaccinia Ankara). This vaccine, however, failed to induce any protective effect (Tameris et al., 2013). Subunit vaccines benefit from novel adjuvants with potent T cell stimulatory capacity without harmful side effects and the elucidation of the genome of *M. tuberculosis* (Del Giudice et al., 2018; Pulendran et al., 2021). Viable vaccines benefit from the advances of genetic modification strategies for mycobacteria (Spertini et al., 2015; Nieuwenhuizen et al., 2017; Kaufmann, 2020; Martín et al., 2021). Also, BCG witnesses a revival because strong evidence was obtained that BCG revaccination of uninfected individuals resulted in prevention of stable infection in almost 50% of study participants (Nemes et al., 2018). The most advanced subunit vaccine is the M72:AS01E, developed by GlaxoSmithKline and now taken over by the Bill and Melinda Gates Medical Research Institute. This subunit vaccine has successfully completed a phase IIb trial which revealed ca. 50% protection against active TB disease over a 3-year observation period in HIV-uninfected, *M. tuberculosis* latently infected adults (Van Der Meeren et al., 2018; Tait et al., 2019).

Viable whole cell vaccines come as two candidates: MTBVAC is a genetically modified *M. tuberculosis* strain with two independent gene deletions which has reached phase IIa clinical assessment (Spertini et al., 2015; Martín et al., 2021). VPM1002 is a genetically modified BCG developed by my own group and then licensed to Vakzine Projekt Management, Germany and Serum Institute of India, Pune, India (Nieuwenhuizen et al., 2017; Kaufmann, 2020). The vaccine is currently in three phase III efficacy trials. One trial (NCT 03152903) assesses PoR in previously drug-treated TB patients; the second trial (Clinical Trial Registry India CTRI/2019/01/017026) determines PoD in household contacts of TB patients under the leadership of the Indian Council of Medical Research (Indian Council Of Medical Research [ICMR], 2019); the third trial determines BCG replacement by VPM1002 in infants (NCT 04351685) because of better safety, efficacy or both as compared to BCG. The Serum Institute of India is the largest vaccine producer on this globe and has already established a high throughput production line with bioreactors, thereby providing the platform for large-scale production and deployment of VPM1002. This is particularly important because currently BCG is produced in canonical liquid cultures which have reached their limits in providing sufficient doses for full vaccine coverage (Kaufmann, 2019a).

## VPM1002: The Most Advanced Live Vaccine

**FIGURE 6:**
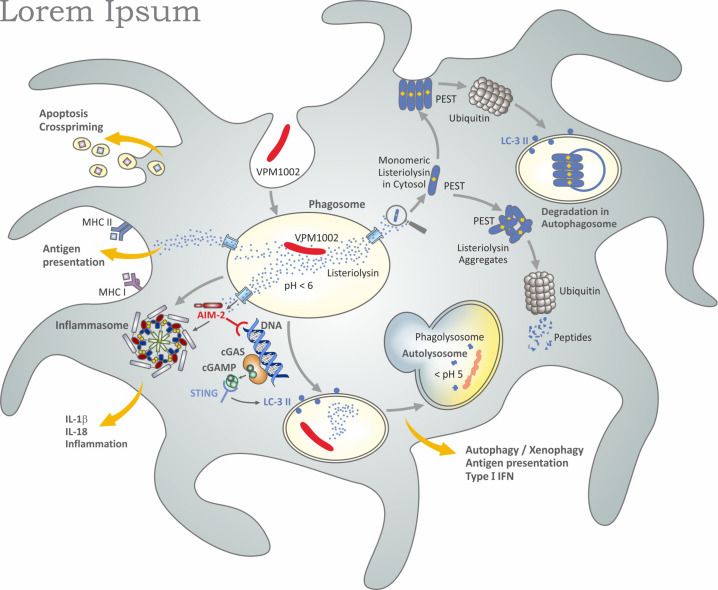
Simplified scheme of potential mechanisms induced by VPM1002 underlying a better safety and efficacy profile over BCG. Figure modified from Kaufmann (2020) and based on published data (Hess et al., 1998; Conradt et al., 1999; Decatur and Portnoy, 2000; Grode et al., 2005; Winau et al., 2006; Farinacci et al., 2012; Saiga et al., 2015; Chen et al., 2018; Nguyen et al., 2019).

VPM1002 is based on the assumption that BCG affords partial protection against TB in neonates. More precisely, it is assumed that BCG induces sufficient immunity to contain *M. tuberculosis* in the lung as primary organ of Mtb infection and thus prevents dissemination to other organs over a prolonged period of time (Nieuwenhuizen et al., 2017; Kaufmann, 2020). BCG has been known to induce long lasting TST positivity after intradermal administration (Albrecht and Paul, 1950; Sjögren, 1982). These earlier studies (see above) are supported by more recent investigations on marked CD4^+^ TH1 cell stimulation in BCG-immunized individuals (Lalor et al., 2011). It was reasoned that this activity, therefore, should be complemented by means which broaden the immune response by inclusion of CD8^+^ CTL (Figure 6; Nieuwenhuizen et al., 2017; Kaufmann, 2020). In order to fulfill this goal, the gene encoding urease C in BCG was replaced by the gene encoding listeriolysin O from *Listeria monocytogenes*. Listeriolysin is a critical virulence factor of *L. monocytogenes* with several intriguing features (Nguyen et al., 2019). As a thiol-activated perforin, it forms pores in the phagolysosomal membrane which leads to the egress of *L. monocytogenes* into the cytosol, where its antigenic peptides are presented in the context of major histocompatibility complex (MHC) I molecules to CD8 T cells. Importantly, the bioactivity of listeriolysin is stringently controlled at an acidic pH around 5.5 (Nguyen et al., 2019). Hence, listeriolysin is only active in an acidified phagosome and not in the extracellular space and blood. Once present in the cytosol, listeriolysin is rapidly degraded because it comprises a so-called PEST sequence (Decatur and Portnoy, 2000; Chen et al., 2018). The PEST sequence, composed of the amino acids proline, glutamine, serine, threonine, is rapidly recognized by the ubiquitination system leading to its prompt degradation and therefore biological inactivation.

BCG is capable of neutralizing the phagosome and hence impairs phagolysosome fusion (Gordon et al., 1980; van der Wel et al., 2007; Ottenhoff and Kaufmann, 2012). Moreover, the neutral pH prevents listeriolysin from being bioactive. The deletion of urease C, however, partly reverses the neutralizing activity of BCG and thus allows for acidification and phagolysosome fusion (Hess et al., 1998; Grode et al., 2005). The bioactive listeriolysin causes perturbation of the phagolysosomal membrane which promotes release of the phagolysosomal content into the cytosol including antigens released by VPM1002 (Pei et al., 2017). Then, listeriolysin in the cytosol can be rapidly degraded by ubiquitination. In contrast to *L. monocytogenes*, VPM1002 apparently does not reach the cytosol itself; only its components are released. Accordingly, VPM1002 stimulates CD8^+^ CTL in addition to CD4^+^ TH1 cells (Conradt et al., 1999; Farinacci et al., 2012; Kaufmann, 2020). Evidence was also presented for a more potent stimulation of antibodies, CD4^+^ TH17 cells and CD4^+^ central memory T cells with specificity for the mycobacterial antigen Ag85B as compared to BCG (Desel et al., 2011; Grode et al., 2013; Vogelzang et al., 2014; Loxton et al., 2017).

In preclinical models, VPM1002 was more rapidly cleared and was less lethal when given intravenously at high doses as compared to BCG providing strong evidence for a better safety profile (Grode et al., 2005; Gengenbacher et al., 2016). Importantly, VPM1002 was found to afford profoundly higher protection against *M. tuberculosis* challenge as compared to BCG in preclinical models. Subsequent studies revealed that the protective efficacy of VPM1002 could be further improved by deleting the anti-apoptotic gene nuoG (Gengenbacher et al., 2016). The nuoG gene encodes an NADH dehydrogenase subunit which impairs apoptosis in host cells (Velmurugan et al., 2007). Evidence suggests that VPM1002 increases apoptosis of its host cells and, thereby, promotes cross-priming leading to improved T-cell stimulation (Winau et al., 2006; Farinacci et al., 2012). Further studies on the intracellular behavior of VPM1002 in comparison to BCG resulted in elevated activation of caspase-1 and caspase-3/7 causing increased inflammasome activation and higher Interleukin (IL)-1β and IL-18 secretion (Saiga et al., 2015). Similarly, VPM1002 was shown to induce LC3-II expression leading to elevated autophagy/xenophagy and, thereby, better antigen presentation as compared to BCG (Saiga et al., 2015). The most likely explanation for these sequelae is the release of DNA into the cytosol which is then sensed by AIM2 and STING which subsequently activate the inflammasome and induce autophagy/xenophagy. Analysis of transcriptomic profiles of lymph-node cells from mice which had been vaccinated with VPM1002 and BCG are consistent with these conclusions since it was found that VPM1002 induced higher transcript expression of genes encoding (pro) IL-18 and (pro) IL-1β as well as STING and genes involved in the Interferon (IFN) type 1 pathway (Saiga et al., 2015).

Studies on host-cell modifications by *M. tuberculosis* revealed a critical role of the RD-1 encoded gene products which are absent from BCG (Mahairas et al., 1996; Behr and Small, 1999; Behr et al., 1999; Pym et al., 2002). Although the RD-1 encoded genes including ESX are totally different from listeriolysin with regard to function and structure, they induce apparently similar down-stream effects as listeriolysin in VPM1002. In both cases, AIM2 sensing of mycobacterial DNA results in inflammasome activation for increased IL-1β and IL-18 production and in STING-mediated activation of LC3-II promoting autophagy/xenophagy, antigen presentation and type 1 IFN induction (Wassermann et al., 2015). Yet, in contrast to listeriolysin, RD-1 encoded gene products are major virulence factors of *M. tuberculosis* (Mahairas et al., 1996; Behr and Small, 1999; Behr et al., 1999; Pym et al., 2002) which are not as rapidly degraded in the cytosol as listeriolysin (Decatur and Portnoy, 2000; Wassermann et al., 2015).

VPM1002 successfully completed phase I trials in young adults in Germany (NCT 00749034) and South Africa (NCT 0113281) and a phase IIa trial in neonates in South Africa (NCT 01479972) (Grode et al., 2013; Loxton et al., 2017). Hence, the vaccine proved safe and immunogenic in neonates and adults. A phase II trial in HIV-exposed and unexposed neonates in South Africa (NCT 02391415) has been completed and results have been submitted for publication. Following these clinical trials, the three phase III trials mentioned above were initiated. The PoR trial (NCT 03152903) comprises 1000 participants each in the VPM1002 and the unvaccinated control group. Participants are being immunized after completed TB drug treatment to determine whether recurrence can be prevented by immunization. Despite apparent cure of active TB by drug treatment, some 10% of individuals are considered to undergo recurrence (either relapse or reinfection) within 1 year after therapy. Recruitment for this trial will likely be completed in 2022. The household contact study (Clinical Trial Registry India CTRI/2019/01/017026) is conceived as a head-to-head testing of VPM1002 and *M. indicus pranii* (an inactivated whole-cell vaccine (Alexander and Turenne, 2015; Sharma et al., 2017)) in addition to unvaccinated controls (Indian Council Of Medical Research [ICMR], 2019). It comprises 4200 household contacts each in the three groups. The recruitment for the trial has been completed and first results are expected toward end of 2021. The priMe study (NCT 04351685) is a phase III multicenter efficacy trial in HIV-exposed and unexposed neonates performed at nine clinical trial sites in five Sub-Saharan countries (Gabon, Uganda, Kenya, Tanzania and South Africa). After some delays, recruitment has started in 2020. The priMe study comprises 3470 participants each in the VPM1002 and the BCG control group. The first clinical endpoint is safety and the second one is PoI by VPM1002 as compared to BCG. In summary, VPM1002 is now undergoing clinical efficacy testing for PoI, PoD and PoR.

## Outlook and Future

With more than a dozen vaccines having entered clinical trial assessments, the future of TB vaccine development looks considerably brighter than before. Yet, the goal remains challenging (Kaufmann et al., 2015). A hundred years after BCG introduction, new approaches have been developed namely virus-vectored vaccines and recombinant *M. tuberculosis* and BCG (Smaill et al., 2013; Tameris et al., 2013; Spertini et al., 2015; Stylianou et al., 2015; Nieuwenhuizen et al., 2017; Kaufmann, 2020; Martín et al., 2021). Also, the subunit vaccine M72:AS01E based on a fusion protein in the novel adjuvant AS01E has delivered promising results (Van Der Meeren et al., 2018; Tait et al., 2019). It is hoped that a new vaccine type based on m-RNA in nanoparticles, which was so successful against COVID-19, will also be designed and tested against TB (Subbarao, 2021). The independent reports on behalf of the WHO “COVID-19: Make it the last pandemic” stated (The Independent Panel, 2021): “Our message for change is clear: No more pandemic. If we fail to take this goal seriously, we will condemn the world to successive catastrophes. The ask is large and challenging but the price is even larger and more rewarding. With so many lives at stake, now is the time to resolve.” In full agreement with this statement for the prevention of future pandemics post-COVID-19, I suggest however that this admonition should not be restricted to newly emerging diseases, but extended to the current threats of HIV/AIDS, malaria, Hepatitis C and of course TB.

## Author Contributions

SK conceived the idea and wrote the manuscript.

## Conflict of Interest

SK is coinventor of the TB vaccine, VPM1002, and coholder of a patent licensed to Vakzine Projekt Management GmbH, Hannover, Germany and sub-licensed to Serum Institute of India Pvt. Ltd., Pune, India. The vaccine is currently undergoing phase III efficacy trial testing.

## Publisher’s Note

All claims expressed in this article are solely those of the authors and do not necessarily represent those of their affiliated organizations, or those of the publisher, the editors and the reviewers. Any product that may be evaluated in this article, or claim that may be made by its manufacturer, is not guaranteed or endorsed by the publisher.
